# Breakthrough candidemia with hematological disease: Results from a single-center retrospective study in Japan, 2009–2020

**DOI:** 10.1093/mmy/myad056

**Published:** 2023-06-13

**Authors:** Ruriko Nishida, Yoshihiro Eriguchi, Noriko Miyake, Yoji Nagasaki, Akiko Yonekawa, Yasuo Mori, Koji Kato, Koichi Akashi, Nobuyuki Shimono

**Affiliations:** Department of Medicine and Biosystemic Science, Kyushu University Graduate School of Medical Science, Fukuoka, Japan; Department of Medicine and Biosystemic Science, Kyushu University Graduate School of Medical Science, Fukuoka, Japan; Department of Medicine and Biosystemic Science, Kyushu University Graduate School of Medical Science, Fukuoka, Japan; Department of Medicine and Biosystemic Science, Kyushu University Graduate School of Medical Science, Fukuoka, Japan; Department of Infectious Disease, National Hospital Organization Kyushu Medical Center, Fukuoka, Japan; Department of Medicine and Biosystemic Science, Kyushu University Graduate School of Medical Science, Fukuoka, Japan; Department of Medicine and Biosystemic Science, Kyushu University Graduate School of Medical Science, Fukuoka, Japan; Department of Medicine and Biosystemic Science, Kyushu University Graduate School of Medical Science, Fukuoka, Japan; Department of Medicine and Biosystemic Science, Kyushu University Graduate School of Medical Science, Fukuoka, Japan; Department of Medicine and Biosystemic Science, Kyushu University Graduate School of Medical Science, Fukuoka, Japan; Center for the Study of Global Infection, Kyushu University Hospital, Fukuoka, Japan

**Keywords:** breakthrough candidemia, hematological disease, hematopoietic stem cell transplantation, echinocandin, *Candida guilliermondii* complex

## Abstract

Breakthrough candidemia (BrC) is a significant problem in immunocompromised patients, particularly those with hematological disorders. To assess the characteristics of BrC in patients with hematologic disease treated with novel antifungal agents, we collected clinical and microbiological information on said patients from 2009 to 2020 in our institution. Forty cases were identified, of which 29 (72.5%) received hematopoietic stem cell transplant (HSCT)-related therapy. At BrC onset, the most administered class of antifungal agents were echinocandins, administered to 70% of patients. *Candida guilliermondii* complex was the most frequently isolated species (32.5%), followed by *C. parapsilosis* (30%). These two isolates were echinocandin-susceptible *in vitro* but had naturally occurring *FKS* gene polymorphisms that reduced echinocandin susceptibility. Frequent isolation of these echinocandin-reduced-susceptible strains in BrC may be associated with the widespread use of echinocandins. In this study, the 30-day crude mortality rate in the group receiving HSCT-related therapy was significantly higher than in the group not receiving it (55.2% versus 18.2%, *P* = .0297). Most patients affected by *C. guilliermondii* complex BrC (92.3%) received HSCT-related therapy and had a 30-day mortality rate of 53.8%; despite treatment administration, 3 of 13 patients had persistent candidemia. Based on our results, *C. guilliermondii* complex BrC is a potentially fatal condition in patients receiving HSCT-related therapy with echinocandin administration.

## Introduction

Invasive fungal infections (IFIs) are life-threatening issues in immunocompromised hosts, particularly patients with hematological disease. Thus, antifungal prophylaxis is regularly used in patients with hematological disease to prevent IFIs.^1–4^ These antifungal treatments can decrease the prevalence of IFI and IFI-related mortality.^5–9^ However, since the 1990s, they have resulted in breakthrough IFIs,^10^, ^11^ which need more attention because new antifungal therapies, such as prophylaxis and novel immunomodulating agents, make diagnosing IFIs difficult.^12–14^ Breakthrough candidemia (BrC) is one of the most important and prevalent breakthrough IFIs. Despite numerous reports on the epidemiology, risk factors, and prognosis of BrC,^15–22^ the epidemiology of IFIs has changed since newer antifungal agents have become available,^23^ particularly those of the echinocandin class.^24^ In addition, emergence of some resistant *Candida* species, such as *C. glabrata* and *C. auris*,^25^, ^26^ and regional differences in resistance rates^27–29^ may influence the epidemiology of BrC. Indeed, several cases of BrC caused by the rare pathogen *C. guilliermondii* complex have been identified in hematological patients at our institution in recent years. *Candida guilliermondii* complex is an uncommon *Candida* species rarely observed to cause fungemia.^30^ Apart from case reports and small surveys,^15^, ^20^, ^31–33^ there is little information regarding BrC involving *C. guilliermondii* complex among patients with hematological disease. Therefore, to clarify the clinical and microbiological information of BrC, particularly associated with respect to the *C. guilliermondii* complex, we conducted a retrospective study on BrC in hematological patients at our institution from 2009 to 2020.

## Materials and methods

### Patients and data collection

We conducted a retrospective analysis on BrC among patients with hematological disease admitted at Kyushu University Hospital (a 1275-bed tertiary teaching hospital in Fukuoka, Japan) between January 2009 and December 2020. We included inpatients undergoing treatments for hematological disease and related conditions, including graft-versus-host disease. Medical records were reviewed for collection of the following clinical information: underlying hematological disease and treatment, clinical symptoms at BrC onset, prior antifungal exposure, antifungal treatment, and outcomes after BrC diagnosis, risk factors for candidemia, including use of corticosteroid and other immunosuppressive agents, central venous catheter (CVC) insertion, abdominal operation, intensive care unit (ICU) stay, use of broad-spectrum antimicrobial agents, and neutropenia. This study was approved by the institutional review board of Kyushu University Hospital (approval no. 22223-00).

### Definitions

Candidemia was defined as the isolation of any *Candida* species from ≥1 blood culture set from a patient with signs and symptoms of infection. BrC was defined as candidemia in patients receiving systemic antifungal agents for ≥3 days before the first positive blood culture.^34^ If the same *Candida* species was detected within 4 weeks in the same patient, then it was considered to be the same episode.^35^, ^36^ Sustained candidemia was defined as candidemia persisting for >2 weeks. Recurrence of candidemia was considered when a blood culture became negative and the same *Candida* species was detected again >4 weeks after the initial detection. Septic shock was defined as sepsis-induced hypotension persisting despite adequate fluid resuscitation.^37^ The serum (1, 3)-beta-D-glucan (BDG) level at onset was defined as the BDG level measured within 3 days before and after the onset of candidemia. The Wako turbidimetric assay (Wako Pure Chemical Industries, Tokyo, Japan) was used to measure the serum BDG level using the cutoff values for positivity recommended by the manufacturer (11 pg/ml).^15^ Neutropenia and severe neutropenia were defined as absolute neutrophil counts of ≤500 cells/µl and ≤100 cells/µl, respectively.^15^ Broad-spectrum antimicrobial agents included carbapenem, fourth-generation cephalosporin, and piperacillin/tazobactam. The choice of antifungal treatment was under the discretion of each physician in all cases.

### Microbiological tests

Blood culture samples were processed using a BacT/Alert system (bioMérieux Japan Ltd, Tokyo, Japan, from January 2009 to March 2013) and a BACTEC FX system (Nippon Becton Dickinson Company Ltd, Tokyo, Japan, from April 2013 to December 2020). Positive samples for yeast according to a gram stain were subcultured on Sabouraud dextrose agar (Nippon Becton Dickinson Company, Ltd, Japan) at 35°C for 48 h. These isolates were initially identified by VITEK 2 YST ID Card (bioMérieux Japan Ltd, from January 2009 to March 2013) and matrix-assisted laser desorption/ionization time-of-flight MALDI VITEK MS (bioMérieux Japan Ltd, from April 2013 to December 2020). Breakthrough *Candida* isolates were stored at −80°C, and molecular identification was conducted by sequencing of the internal transcribed spacer region according to the CLSI MM18-A protocol.

An antifungal susceptibility test was performed using broth microdilution methods with a commercial kit ASTY (Kyokuto Pharmaceutical Industrial, Tokyo, Japan) following the manufacturer’s instructions. For the four species (*C. albicans, C. glabrata, C. krusei*, and *C. parapsilosis*), minimal inhibitory concentration (MIC) results were interpreted following the Clinical and Laboratory Standards Institute (CLSI) clinical breakpoints (CBPs) according to the CLSI M27-S4 standard. For agents without species-specific CBPs, epidemiological cutoff values (ECVs) were applied.^38^ The MIC interpretation for *C. nivariensis* was determined according to CLSI standards for *C. glabrata*. In this study, we defined non-susceptible strains as those whose susceptibility test results were determined other than susceptible by CBPs and non-wild type by ECVs. *Candida parapsilosis* ATCC 22019 was tested as a quality control for every new lot.

In addition, we performed screening of mutations in hot spot regions of *FKS* genes. The *FKS1* hot spot 1 (HS1) and hot spot 2 (HS2) regions were sequenced for all breakthrough *Candida* isolates. In addition, *FKS2* HS1 and HS2 regions were also analyzed in *C. glabrata*. Polymerase chain reaction amplification was conducted using two sets of primers previously described (https://doi.org/10.3030/642095).^25^, ^39^

### Statistical analysis

Continuous data are expressed as the median and interquartile range (IQR), and categorical data are described by the count and percentage. The Mann–Whitney *U*-test was used to compare continuous variables, and the Fisher’s exact test was used to compare categorical variables. The survival curves at 30 days were calculated using the Kaplan–Meier method, and differences between curves were evaluated using the log-rank test. A *P*-value < .05 was considered statistically significant. All statistical tests were conducted using EZR.^40^

## Results

### Characteristics of BrC in hematological patients

From January 2009 to December 2020, 48 episodes of fungemia (44 candidemia, 2 fusariosis, and 2 trichsporonemia) were detected in patients with hematological disease at Kyushu University Hospital. A total of 40 cases of candidemia in 39 patients met the definition of BrC. Only one patient developed two episodes of BrC (due to *C. lusitaniae* and *C. parapsilosis*, respectively) during the study period. The clinical characteristics of the 40 episodes are shown in Table 1. The median age at BrC onset was 55.5 years (IQR, 44–64 years). Of the underlying hematological diseases, myeloid and lymphatic malignancies accounted for 47.5% and 50% of cases, respectively. Of all, 29 cases (29/40, 72.5%) occurred during hematopoietic stem cell transplantation (HSCT)-related treatment; 14 patients (14/40, 35.0%) had received multiple HSCT. In the case of HSCT-related BrC, 18 cases (18/40, 45.0%) occurred during the HSCT conditioning or pre-engraftment period, whereas 11 other patients (11/40, 27.5%) developed BrC during the post-engraftment period. The other 11 BrC episodes developed during HSCT non-related treatment (eight cases of chemotherapy for lymphoma and three of induction therapy for acute myeloid leukemia, respectively). The prevalence of risk factors for candidemia in our BrC patients is shown in Table 1. The presence of a CVC, total parenteral nutrition, and use of broad-range antimicrobial agents were observed in almost all cases (40/40, 100%; 36/40, 90%; and 39/40, 97.5%, respectively). Over half of the patients had immunosuppressive therapy, neutropenia, and mucositis.

**Table 1.** Clinical characteristics of 40 BrC cases.

Characteristic	Result (*n* = 40)
Age, median years (IQR)	55.5 (44–64)
Gender, male	23 (57.5%)
Underlying hematological disease	
Myeloid malignancy	19 (47.5%)
Lymphoid malignancy	20 (50%)
Other	1 (2.5%)
Treatment of hematological disease	
HSCT related	29 (72.5%)
BrC onset after HSCT, median days (IQR)	50 (16–106)
Conditioning	5 (12.5%)
Pre-engraftment	13 (32.5%)
Post-engraftment	11 (27.5%)
GVHD	5 (12.5%)
Relapse	3 (7.5%)
Other supportive therapy	3 (7.5%)
Prior transplantation	14 (35%)
HSCT non-related	11 (27.5%)
Leukemia induction therapy/Other chemotherapy	3/8 (7.5%/20%)
Risk factors for candidemia	
Immunosupression	
Neutropenia/severe neutropenia	23/20 (57.5%/50%)
Systemic corticosteroids	29 (72.5%)
Other immunosuppressive agents	26 (65%)
Central venous catheter/total parenteral nutrition	40/36 (100%/90%)
Broad-spectrum antimicrobial agents	39 (97.5%)
ICU stay (previous 30 days)	10 (25%)
Abdominal operation (previous 30 days)	1 (2.5%)
Mucositis	21 (52.5%)
Complications	
Endophthalmitis	1 (2.5%)
Meningitis	1 (2.5%)
Serum (1,3)-beta-D-glucan (≥11 pg/μl)	20 (50%)
Death within 30 days after BrC onset	18 (45%)

Data are the number. (%) of cases, unless otherwise indicated.

BrC, breakthrough candidemia; IQR, interquartile range; HSCT, hematopoietic stem cell transplantation; GVHD, graft versus host disease; ICU, intensive care unit.

### Isolated *Candida* species

The *Candida* species causative of BrC are shown in Table 2. *Candida guilliermondii* complex (13/40, 32.5%) was the most frequent isolate, followed by *C. parapsilosis* (12/40, 30%), *C. krusei* (4/40, 10%), *C. albicans* (3/40, 7.5%), *C. lusitaniae* (3/40, 7.5%), *C. nivariensis* (2/40, 5%), *C. glabrata* (1/40, 2.5%), *C. kefyr* (1/40, 2.5%), and *C. rugosa* (1/40, 2.5%).

**Table 2.** *Candida* species distribution.

Species	*n *= 40
*C. guilliermondii* complex	13 (32.5%)
*C. parapsilosis*	12 (30%)
*C. krusei*	4 (10%)
*C. albicans*	3 (7.5%)
*C. lusitaniae*	3 (7.5%)
*C. nivariensis*	2 (5%)
*C. glabrata*	1 (2.5%)
*C. kefyr*	1 (2.5%)
*C. rugosa*	1 (2.5%)

### Antifungal therapy at BrC onset

Tables 3 and 4 show the antifungal therapy at BrC onset. Micafungin (MCF, 15 cases), caspofungin (CPF, 9 cases), liposomal amphotericin B (L-AMB, 6 cases), fluconazole (FLC, 3 cases), voriconazole (VCZ, 3 cases), CPF plus L-AMB (2 cases), CPF plus FLC (1 case), and CPF plus VCZ (1 case) were administered when BrC occurred. The most administered class of antifungal agents were echinocandins, which were administered in 70% of all cases. The next most common were L-AMB (25%), and azoles (20%). Six patients (15%) received antifungal agents (FLC, 3 patients; MCF, 3 patients) at lower than therapeutic doses. The remaining 34 patients (85%), including one case who received a reduced corrected dose of antifungal medication due to hepatic dysfunction, developed BrC despite receiving therapeutic doses. The median time of prior antifungal treatment was 31 days (IQR, 19–44.5 days).

**Table 3.** Antifungal therapy at the onset of BrC.

Antifungal therapy	*n* = 40
Single antifungal agent therapy	36 (90%)
Echinocandin	24 (60%)
MCF	15 (37.5%)
CPF	9 (22.5%)
Azole	6 (15%)
FLC	3 (7.5%)
VCZ	3 (7.5%)
L-AMB	6 (15%)
Combination therapy	4 (10%)
Dose of antifungal agents^a^	
Low dose	6 (15%)
Therapeutic dose	34 (85%)
Duration of antifungal therapy, median days (IQR)	31 (19–45)

Data are the number. (%) of cases, unless otherwise indicated.

BrC, breakthrough candidemia; MCF, micafungin; CPF, caspofungin; FLC, fluconazole; VCZ, voriconazole; L-AMB, liposomal amphotericin B; IQR, interquartile range.

a The therapeutic dose refers to FCZ ≥ 400 mg/day, MCF ≥ 100 mg/day, CAS ≥ 50 mg/day, and L-AMB ≥ 2 mg/kg/day for each drug. The low dose was less than the therapeutic dose. Dose reduction due to liver dysfunction is included in the therapeutic dose.

**Table 4.** Summary of BrC by class of prior antifungal agents.

No.	Hematological disease	HSCT	Prior antifungal therapy		*Candida* species	MIC (μg/ml)					Susceptibility for prior antifungal agents	Fksp amino acid substitution				β-D-glucan (≥11 pg/μl)	Treatment after BrC diagnosis	Outcome at 30 days
			Antifungal agents	Duration (days)		FLC	VCZ	MCF	CPF	AMB		Fks1 HS1	Fks1 HS2	Fks2 HS1	Fks2 HS2			
1	ML	–	FLC 100 mg	12	*C. albicans*	0.5	≤0.015	0.03	**0.5**	1	S	WT	WT	…	…	+	VCZ	Alive
2	ALL	+	F-FLC 200 mg	8	*C. albicans*	0.5	≤0.015	≤0.03	**0.5**	0.5	S	WT	WT	…	…	+	L-AMB	Deceased
3	AML	–	FLC 200 mg	21	*C. krusei*	**≥128**	**1**	0.25	**0.5**	1	non-WT	WT	WT	…	…	+	MCF → MCF + L-AMB	Alive
4	AML	+	VCZ 400 mg	91	*C. krusei*	**>64**	**>8**	0.125	**1**	1	NS	WT	WT	…	…	+	VCZ + MCF	Deceased
5	AML	+	VCZ 400 mg	105	*C. lusitaniae*	**≥128**	**4**	0.06	**0.5**	0.5	non-WT	WT	WT	…	…	+	Died before BrC diagnosis	Deceased
6	AML	+	VCZ 400 mg	58	*C. guilliermondii*	**≥128**	**≥16**	0.5	1	0.5	non-WT	WT	WT	…	…	–	CPF + L-AMB	Deceased
7	ML	+	MCF 75 mg ^d^	14	*C. glabrata*	4	0.25	**1**	4	0.5	NS	S629P	WT	WT	WT	–	MCF + L-AMB	Deceased
8	MDS	+	MCF 300 mg	32	*C. albicans*	1	≤0.015	**1**	4	0.5	NS	S645P	WT	…	…	+	L-AMB	Alive
9	MDS	+	MCF 75 mg	27	*C. guilliermondii*	**64**	**1**	0.25	1	0.5	S	WT	WT	…	…	**-**	L-AMB	Deceased
10	AML-MRC	+	MCF 150 mg	30	*C. guilliermondii*	**16**	**0.5**	0.06	0.5	0.5	S	WT	WT	…	…	–	L-AMB	Deceased
11	AML	+	MCF 50 mg	42	*C. guilliermondii*	8	0.25	0.25	1	0.5	S	WT	WT	…	…	+	L-AMB	Alive
12	AML	+	MCF 50 mg	38	*C. guilliermondii*	8	0.25	0.25	0.5	1	S	WT	WT	…	…	–	L-AMB	Deceased
13	AML	+	MCF 150 mg	54	*C. guilliermondii*	4	0.125	0.125	0.5	0.5	S	WT	WT	…	…	–	L-AMB	Alive
14	MDS	+	MCF 150 mg	63	*C. parapsilosis*	2	0.06	1	1	0.5	S	WT	WT	…	…	+	L-AMB	Deceased
15	ML	+	MCF 150 mg	31	*C. parapsilosis*	1	0.03	0.5	1	1	S	WT	WT	…	…	+	VCZ → L-AMB	Alive
16	ALL	+	MCF 150 mg	79	*C. parapsilosis*	2	0.06	2	2	0.5	S	WT	WT	…	…	–	L-AMB → FLC	Alive
17	ML	+	MCF 150 mg	63	*C. parapsilosis*	1	0.03	0.5	1	1	S	WT	WT	…	…	+	L-AMB	Alive
18	ML	–	MCF 150 mg	23	*C. parapsilosis*	1	0.03	0.5	1	0.5	S	WT	WT	…	…	–	L-AMB	Alive
19	AML	–	MCF 150 mg	21	*C. parapsilosis*	1	0.03	0.5	1	1	S	WT	WT	…	…	–	MCF (continued same drug)	Alive
20	ML	+	MCF 150 mg	63	*C. lusitaniae*	**2**	≤0.015	0.25	**0.5**	1	S	WT	WT	…	…	–	L-AMB → VCZ	Deceased
21	ML	–	MCF 150 mg	10	*C. krusei*	**≥128**	**1**	0.125	**1**	1	S	WT	WT	…	…	–	MCF (continued same drug)	Alive
22	ALL	+	CPF 50 mg	32	*C. kefyr*	0.5	≤0.015	**1**	**2**	1	non-WT	F651S	WT	…	…	–	L-AMB + VCZ	Alive
23	AML	–	CPF 50 mg	20	*C. parapsilosis*	0.5	≤0.015	1	1	1	S	WT	WT	…	…	–	L-AMB	Alive
24	AML	+	CPF 50 mg	91	*C. parapsilosis*	**8**	0.125	1	1	0.5	S	WT	WT	…	…	+	L-AMB → VCZ	Alive
25	ML	–	CPF 50 mg	10	*C. parapsilosis*	1	0.03	0.5	0.5	0.5	S	WT	WT	…	…	+	CPF + L-AMB → CPF + VCZ	Deceased
26	ML	–	CPF 50 mg	3	*C. parapsilosis*	2	0.03	0.25	0.5	0.5	S	WT	WT	…	…	+	L-AMB → FLC	Alive
27	ATL	–	CPF 50 mg	54	*C. parapsilosis*	1	≤0.015	0.5	1	0.5	S	WT	WT	…	…	–	CPF + FLCZ	Alive
28	ML	+	CPF 50 mg	21	*C. parapsilosis*	1	0.03	0.5	1	1	S	WT	WT	…	…	–	L-AMB	Alive
29 ^a^	MPN	+	CPF 50 mg	26	*C. guilliermondii*	8	**0.5**	2	1	1	S	WT	WT	…	…	+	CPF + L-AMB → CPF + VCZ	Alive
30 ^b^	ML	–	CPF 50 mg	40	*C. guilliermondii*	8	0.25	0.125	0.25	0.5	S	WT	WT	…	…	–	L-AMB	Deceased
31	AML	+	L-AMB 2.5 mg/kg	42	*C. krusei*	64	0.5	0.125	**0.5**	1	WT	WT	WT	…	…	+	MCF + L-AMB	Deceased
32	MM	+	L-AMB 2 mg/kg	14	*C. rugosa*	4	0.06	8	16	1	Inconclusive^e^	WT	WT	…	…	–	MCF + L-AMB → MCF + VCZ	Deceased
33	ML	+	L-AMB 2.5 mg/kg	5	*C. guilliermondii*	0.5	0.25	0.25	0.5	1	WT	WT	WT	…	…	–	Died before BrC diagnosis	Deceased
34	ML	–	L-AMB 3 mg/kg	32	*C. lusitaniae*	**2**	0.03	0.5	0.25	1	WT	WT	WT	…	…	+	MCF + L-AMB → MCF + VCZ	Alive
35 ^c^	AML	+	L-AMB 2.5 mg/kg	44	*C. guilliermondii*	4	**1**	0.5	1	1	WT	WT	WT	…	…	–	MCF + VCZ	Alive
36	AML-MRC	+	L-AMB 2 mg/kg	20	*C. guilliermondii*	**16**	**0.5**	0.125	0.5	0.25	WT	WT	WT	…	…	–	L-AMB → L-AMB + CPF	Alive
37	ML	+	CPF 50 mg + VRC 100 mg	35	*C. nivariensis*	**≥128**	**4**	0.06	**0.5**	1	NS/non-WT	WT	WT	…	…	+	CPF + VCZ → L-AMB	Deceased
38	ALL	+	CPF 50 mg + FLC 200 mg	7	*C. guilliermondii*	**16**	**0.5**	0.125	0.5	1	S/non-WT	WT	WT	…	…	+	L-AMB	Deceased
39	ML	+	CPF 50 mg + L-AMB 1 mg/kg	4	*C. nivariensis*	2	0.06	**0.5**	**2**	0.5	NS/WT	WT	WT	…	…	+	CPF + L-AMB	Deceased
40	DBS	+	CPF 50 mg + L-AMB 3 mg/kg	8	*C. guilliermondii*	1	≤0.015	0.5	0.5	1	S/WT	WT	WT	…	…	+	CPF + VCZ	Alive

In the MIC columns, shaded areas indicate the antifungal agents administrated at the onset of breakthrough candidemia. MIC values higher than the CBPs or ECVs are shown in bold. The CBPs and ECVs are listed in Table S1. HSCT, hematopietic stem cell transplantation; MIC, minimal inhibitory concentration; FLC, fluconazole; F-FLC, fosfluconazole; VCZ, voriconazole; MCF, micafungin; CPF, caspofungin; AMB, amphotericin B; L-AMB, liposomal amphotericin B; ALL, acute lymphoblastic leukemia; AML, acute myeloid leukemia: AML-MRC, AML with myelodysplasia-related changes; MDS, myelodysplastic syndromes; ML, malignant lymphoma; MPN, myeloproliferative neoplasms; DBS, Diamond-Blackfan syndrome; S, susceptible; NS, non-susceptible; WT, wild-type; non-WT, non-wild type; X##X = amino acid, position, amino acid substitute; ‘… ’ = not tested.

a, b, and c Sustained candidemia (≥14 days) cases.

c Recurrence of candidemia case.

d Dose reduction due to liver dysfunction.

e No criteria to determine drug susceptibility to *C. rugosa*.

### Antifungal susceptibility to previously administered agents

Six of eight BrC cases (75%) under azole therapy were caused by *Candida* species not susceptible to the preceding azoles *in vitro* (Table 4). The remaining two patients received low-dose FLC (100 or 200 mg/day, Table 4); in both cases, FLC-sensitive *C. albicans* was isolated. On the other hand, most BrCs during echinocandin treatment (23/28, 82.1%) were caused by *Candida* species susceptible to the echinocandins used; all eight BrCs during L-AMB administration were due to AMB-sensitive *Candida* species. The trend in susceptibility of BrC isolates to the prior drug was that azole-resistant strains were more common in the azole pre-treated group, whereas isolates from echinocandin pre-treated cases and L-AMB pre-treated cases were often sensitive to the prior drug, consistent with previous reports.^15–17^, ^19^, ^20^, ^32^, ^41^

The susceptibility to prior echinocandin treatment depends on *Candida* species. All isolates of the *C. guilliermondii* complex (9 isolates), *C. parapsilosis* (12 isolates), *C. krusei* (1 isolate), and *C. lusitaniae* (1 isolate) were susceptible to echinocandins, whereas *C. nivariensis* (2 isolates), *C. albicans* (1 isolate), *C. glabrata* (1 isolate), and *C. kefyr* (1 isolate) were not. Since reduced susceptibility to echinocandin involves amino acid substitutions in the hot spot region of the *FKS* protein, the catalytic subunit of the 1,3-β-D-glucan synthase,^25^, ^42–45^ we evaluated the presence of *FKS* gene mutations in isolates. Each strain of *C. glabrata, C. albicans*, and *C. kefyr* isolated during echinocandin therapy showed a mutation in the HS1 region of *FKS1* and non-susceptibility to prior echinocandins (Table 4). All *C. guilliermondii* complex and *C. parapsilosis* isolates harbored naturally occurring polymorphisms in the *FKS1* region, showing a tendency toward higher echinocandin MICs than wild-type *C. albicans*, as previously reported.^42–45^

### Treatments and outcomes after BrC diagnosis

A detailed description on treatments and outcomes is shown in Table 4. A total of 38 cases (95%) were diagnosed with BrC; two (5%) patients died before the diagnosis of breakthrough infection. The crude mortality rates at days 14 and 30 were 32.5% and 45.0%, respectively. The median time from BrC to death within 30 days was 9.5 days (IQR, 3–15 days). Thirty-three cases (86.8%) changed the antifungal therapy after BrC, while five (13.2%) continued with the prior antifungal agents. One case treated with VRC died before BrC diagnosis; the isolates were later confirmed to be resistant to VRC. The prior azole treatments of the other five cases were changed to therapies comprising susceptible antifungal agents; however, three (60%) patients died within 30 days after BrC. In addition, 21 (87.5%) BrC patients with prior echinocandin monotherapies received L-AMB-based regimens, including combination therapies; of those, 1/3 died within 30 days of diagnosis. Two patients (8.3%) continued with their previous MCF treatment because of the good susceptibility and clinical course. The last case (4.2%) was continuously treated with CPF and received FLC after diagnosis, resulting in a favorable outcome. Five of six prior L-AMB treatment cases (83.3%) were added or changed to echinocandin agents with or without VRC after diagnosis; two of whom died. One case receiving L-AMB treatment died before BrC diagnosis, although the isolate was sensitive to amphotericin B. One BrC case receiving combination therapy was treated with L-AMB monotherapy after the prior treatment proved ineffective, with a fatal consequence. Two BrC cases in which one of the two prior antifungal drugs was ineffective were either switched to another class of agents or continued on the prior medication, but both died. The remaining BrC case on combination therapy changed treatment from L-AMB to VCZ on a decision of ineffective previous therapy and survived, although both previous medications were susceptible to the isolate. The patients that developed BrC during combination therapy had a worse prognosis, with 3 out of 4 patients dying, possibly because they were more severely ill, given that all patients received HSCT-related therapy.

### Features of *C. guilliermondii* and *C. parapsilosis* breakthrough infection

Since the *C. guilliermondii* complex and *C. parapsilosis* were the two dominant species in this study (Table 2), we focused on the characteristics of breakthrough infection caused by these two species (Table 5). Patient age and sex were similar in both *C. guilliermondii* complex and *C. parapsilosis* BrC cohorts. Myeloid malignancy was the dominant hematological disease in the *C. guilliermondii* complex group (69.2%), while lymphoid malignancy was most common in the *C. parapsilosis* group (66.7%). Most patients in the *C. guilliermondii* complex cohort (12 cases, 92.3%) received HSCT-related therapy. In contrast, the *C. parapsilosis* cohort had significantly fewer patients receiving HSCT treatment (6 cases, 50%, *P* = .03). Among the risk factors for candidemia, the rates of administration of systemic corticosteroid and CVC insertion were similar, whereas ICU stay and neutropenia were slightly more frequent in the *C. guilliermondii* complex group (46.2% versus 16.7%, *P* = .202; 76.9% versus 50.0%, *P* = .226, respectively). Most patients of the *C. parapsilosis* cohort presented with fever at BrC onset compared to the *C. guilliermondii* complex (91.7% versus 46.2%, *P* = .032). No patient in the *C. parapsilosis* cohort sustained or relapsed from candidemia, while 3 (23.1%) had a persisting candidemia for >14 days in the *C. guilliermondii* complex despite immediate CVC removal after BrC diagnosis (No. 29, 30, and 35 cases, 20 days, 23 days, and 37 days, respectively, Table 4). Moreover, the No. 35 case relapsed after one month. The incidence of septic shock at BrC onset was similar in both *C. guilliermondii* complex and *C. parapsilosis* groups (15.4% and 16.7%, respectively), as was the presence of disseminated infections (7.6% and 8.3%, respectively). The positive rate of serum BDG was 50% in the *C. parapsilosis* cohort, same as for all BrC cases in this study (Table 1), while that in the *C. guilliermondii* complex cohort was lower (30.8%).

**Table 5.** Feature comparison of *C. guilliermondii* complex and *C. parapsilosis* breakthrough infection.

	*C. guilliermondii* complex (*n* = 13)	*C. parapsilosis* (*n *= 12)	*P*-value
**Demographics**			
Median age (IQR)	58 (47–64)	52 (43–63)	–
Gender, male	8 (61.5%)	7 (58.3%)	–
**Underlying hematological disease and treatment**			
Myeloid malignancy	9 (69.2%)	4 (33.3%)	
Lymphoid malignancy	3 (23.1%)	8 (66.7%)	
Other	1 (7.7%)	0 (0%)	
HSCT-related therapy	12 (92.3%)	6 (50%)	0.03
**Risk factors of candidemia**			
Systemic corticosteroids	10 (76.9%)	7 (58.3%)	0.411
CVC/TPN	13/11 (100%/84.6%)	12/11 (100%/91.7%)	–
ICU stay (previous 30 days)	6 (46.2%)	2 (16.7%)	0.202
Neutropenia	10 (76.9%)	6 (50%)	0.226
Median neutrophil at onset (/µl, IQR)	5 (0–470)	714 (25–2475)	0.112
Mucositis	6 (46.2%)	6 (50%)	1
**Clinical features**			
Fever at onset	6 (46.2%)	11 (91.7%)	0.032
Sustained candidemia (≥14 days)	3 (23.1%)	0 (0%)	0.22
Recurrence of candidemia	1 (7.7%)	0 (0%)	1
Septic shock at onset	2 (15.4%)	2 (16.7%)	1
Disseminated infection	1 (7.7%)	1 (8.3%)	1
Serum (1,3)-beta-D-glucan positive	4 (30.8)	6 (50%)	0.428
Deaths within 30 days	7 (53.8%)	2 (16.7%)	0.0968
**Prior antifungal agent**			
Monotherapy	11 (84.6%)	12 (100%)	–
Echinochandin	7 (53.8%)	12 (100%)	–
Azole	1 (7.7%)	0 (0%)	–
L-AMB	3 (23.1%)	0 (0%)	–
Combination therapy	2 (15.4%)	0 (0%)	–
Median duration days (IQR)	30 (20–42^)^	27 (21–63)	0.531
**Susceptibility to antifungal agents**			
MIC_50_/MIC_90_ (µg/ml, range)			
MCF	0.25/0.5 (0.06–2)	0.5/1 (0.5–2)	
CPF	0.5/1 (0.25–1)	1/1 (0.5–2)	
FLC	8/64 (0.5–≥128)	1/2 (0.5–8)	
VRC	0.5/1 (≤0.015–≥16)	0.03/0.06 (≤0.015–0.125)	
AMB	0.5/1 (0.25–1)	0.5/1 (0.5–1)	
Non-susceptible for prior antifungal agent (%)^a^	2 (15.4)	0 (0)	0.48

Data are the number. (%) of cases, unless otherwise indicated.

IQR, interquartile range; HSCT, hematopietic stemcell transplantation; ICU, intensive care unit; CVC, central venous catheter; TPN, total parenteral nutrition; L-AMB, liposomal amphotericin B; FLC, fluconazole; VCZ, voriconazole; MCF, micafungin; AMB, amphotericin B; MIC, minimum inhibitory concentration.

a In the case of the combination therapy, non-susceptible include the cases that were not susceptible for one or more of the prior agents.

Echinocandin monotherapy was the most frequent prior treatment of both *C. parapsilosis* and *C. guilliermondii* complex BrC; in particular, all 12 *C. parapsilosis* BrC episodes occurred while using echinocandins. On the other hand, the *C. guilliermondii* complex was isolated in 13 patients treated with all classes of antifungal agents (MCF, 5; CPF, 2; L-AMB, 3; VCZ, 1; CPF plus VCZ, 1; and CPF plus L-AMB, 1; respectively). Median time of prior antifungal agent use was similar between groups (*C. guilliermondii* complex, 30 days; *C. parapsilosis*, 27 days, respectively).

All *C. parapsilosis* isolates were susceptible to prior echinocandins, whereas two *C. guilliermondii* complex isolates were non-susceptible to prior azole agents. Comparing the MIC azole values between *C. guilliermondii* complex and *C. parapsilosis*, MIC_50_ values were higher in the *C. guilliermondii* complex (FLC, 8 µg/ml versus 1 µg/ml; VRC 0.5 µg/ml versus 0.03 µg/ml, respectively) as were MIC_90_ values (FLC, 64 µg/ml versus 2 µg/ml; VRC 1 µg/ml versus 0.06 µg/ml, respectively).

**Figure 1.**
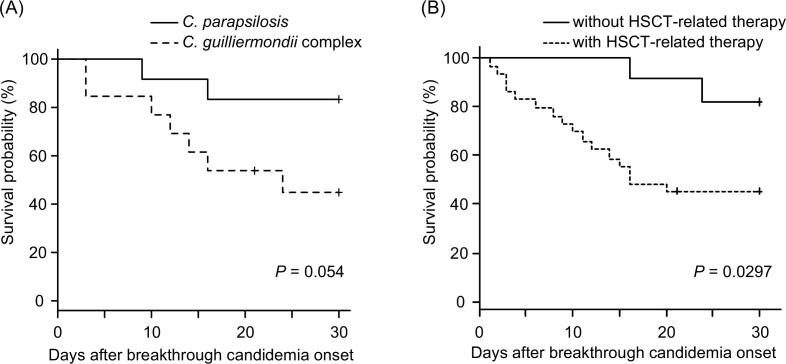
Kaplan–Meier survival curves of patients with BrC. (A) Curves of patients with BrC due to *C. guilliermondii* complex and *C. parapsilosis*. (B) Curves of patients receiving or not receiving HSCT-related therapy. *P* values were calculated using the log-rank test.

The survival curves at 30 days of both groups, calculated using the Kaplan–Meier method (Fig. 1a), show that the 30-day survival rate of the *C. parapsilosis* group was higher—but not significantly different—than that of the *C. guilliermondii* complex (83.3%, 46.2%, respectively, *P* = .054).

### Mortality in BrC patients with and without HSCT-related therapy

The high mortality of *C. guilliermondii* BrC (53.8%) was associated with a higher rate of HSCT treatment (92.3%) than in the *C. parapsilosis* cohort (Fig. 1a and Table 5). Furthermore, all BrC cases observed during combination therapy also received HSCT-related treatment. Given the high mortality rate (75%), to determine whether HSCT was relevant to the BrC outcomes, we evaluated the impact of HSCT-related therapy on BrC survival. BrC patients receiving HSCT-related treatment had a significantly lower survival at 30 days than the non-HSCT cohort (Fig. 1b, 44.8% versus 81.8%, *P* = .0297).

## Discussion

In this study, we described the clinical and microbiological features of 40 BrC cases in patients with hematologic diseases at a single tertiary institution. The most frequent isolate was the *C. guilliermondii* complex (32.5%), and the second was *C. parapsilosis* (30.0%). To the best of our knowledge, the unfavorable characteristics of the *C. guilliermondii* complex BrC in hematological patients are highlighted for the first time in this study.

*Candida guilliermondii* complex is an uncommon yeast, responsible for only 1.4% of all candidemia cases.^30^ This proportion depends on the geographic region, with relatively higher rates in Asia (1.8%–2.6%) and Latin America (3.7%).^30^, ^31^, ^46^ The proportion of *C. guilliermondii* complex is slightly higher when it is the causative agent of BrC (4.8%–15%).^15, 16, 20, 34^ Most *C. guilliermondii* complex candidemia has been reported in patients with cancer, especially those with hematological malignancies.^15^, ^16^, ^31^, ^34^, ^47–49^ This might explain why *C. guilliermondii* complex was found in most patients in our study. However, our data showed a higher incidence than previously reported (4.8%–15%),^15^, ^16^, ^20^, ^34^ which may be due to regional characteristics or other factors. The Japanese national surveillance data on candidemia (2010–2019) show an increasing frequency of infection with *C. guilliermondii* since 2014.^49^ In addition, the incidence rate of candidemia caused by *C. guilliermondii* complex and *C. parapsilosis* in Japanese hospitals providing HSCT was significantly higher than that in other hospitals.^49^ In our study, *C. guilliermondii* complex BrC with hematological disease was detected in 2011 (Fig. S1). Although there was no clear upward trend in *C. guilliermondii* complex BrC, the combined proportion of *C. guilliermondii* complex and *C. parapsilosis* BrC showed an increasing trend from 2011 onwards. The proportion of *C. guilliermondii* complex in BrC with hematological disease in this study was higher than that of *C. guilliermondii* complex in all candidemia in our hospital over the same period, 2009–2020. Thus, our single-center data are consistent with the general trend in Japan.

In our study, breakthrough infections with *C. guilliermondii* complex tended to be more common in HSCT patients (92.3%) and showed a high mortality rate (53.8%); there were three cases (23.1%) of *C. guilliermondii* complex candidemia lasting >2 weeks, of which one case relapsed after one month, suggesting that these refractory fungemia cases might be related to severe host conditions, especially HSCT, and that the breakthrough infection may not be the direct cause of death. Indeed, the mortality rate for BrC in HSCT recipients was significantly higher than in the non-HSCT cohort (*P* = .0297, Fig. 1b). *Candida guilliermondii* complex BrC in hematological patients may be a potentially fatal condition, including critical underlying disease. A previous report^50^ showed that *C. guilliermondii* complex candidemia was more frequently persistent than that of *C. albicans*; moreover, patients with *C. guilliermondii* complex candidemia had usually a severe underlying disease but a lower mortality rate and less severe clinical presentations than those with *C. albicans*. This is mostly consistent with our results, except for the lower mortality rate; the reason for the higher mortality rate in our patients could be that 92.3% were receiving HSCT-related treatment and may have had more severe underlying conditions.

Previously, *C. albicans* was the most frequently isolated species, but the widespread FLC use has led to increased isolation rate of relatively azole-resistant *Candida* species, such as *C. glabrata* and *C. krusei*.^11^, ^17^, ^51–56^ Over the last decade, *C. parapsilosis* has been reported as the most common causative organism (41%–56%) in BrC observed during echinocandin administration, especially in hematological patients.^15^, ^57^, ^58^ In this study, echinocandins were the most common antifungal agents administered at BrC onset; 60% of all patients received echinocandin monotherapy and 10% combination therapy including echinocandins (Tables 2 and 4). Furthermore, *C. parapsilosis* was the second most common pathogen causing BrC (30%) (Table 3). The high frequency of BrC caused by the *C. guilliermondii* complex and *C. parapsilosis* in our study could be attributed to their intrinsically low echinocandin susceptibility and the high frequency of echinocandin use. In this study, all cases of BrC due to *C. parapsilosis* developed during echinocandin administration as did 9 of 13 cases of BrC due to the *C. guilliermondii* complex; the remaining four cases with the *C. guilliermondii* complex received echinocandins as prior therapy within the previous 60 days. All *C. guilliermondii* complex and *C. parapsilosis* isolates harbored naturally occurring polymorphisms in the *FKS1* region and showed a tendency toward higher echinocandin MICs than wild-type *C. albicans*, as previously reported.^42–45^ All these isolates were echinocandin-sensitive according to CLSI M60-Ed2 criteria. However, since they caused BrC, there might be no relation between the efficacy *in vivo* and MIC *in vitro*, particularly in hematological patients. All *C. guilliermondii* complex isolates in this study had the naturally occurring L633M and T634A substitutions as intrinsic hot spot mutations in *FKS1*, consistent with previous reports.^44^ The positive BDG rate was low in the *C. guilliermondii* complex BrC, 4 out of 13 cases (30.8%); thus, these *FKS1* intrinsic mutations and the use of antifungal drugs such as echinocandin could potentially affect BDG synthesis.^59^ Future research is needed to clarify this point.

This study had some limitations. It was retrospectively conducted at a single tertiary-care teaching hospital. Thus, its results may not be generalizable to other institutions. In addition, the number of cases was relatively small. More cases need to be accumulated to evaluate prognosis according to *Candida* species and to analyze the clinical characteristics of BrC patients who received HSCT-related therapy.

## Supplementary Material

myad056_Supplemental_Files
