# Supplementary figures and images for: Breakthrough candidemia with hematological disease: Results from a single-center retrospective study in Japan, 2009–2020
Source: Med Mycol. 2023 Jun 13;61(6):myad056. doi: 10.1093/mmy/myad056 (PMC10294639; doi:10.1093/mmy/myad056)

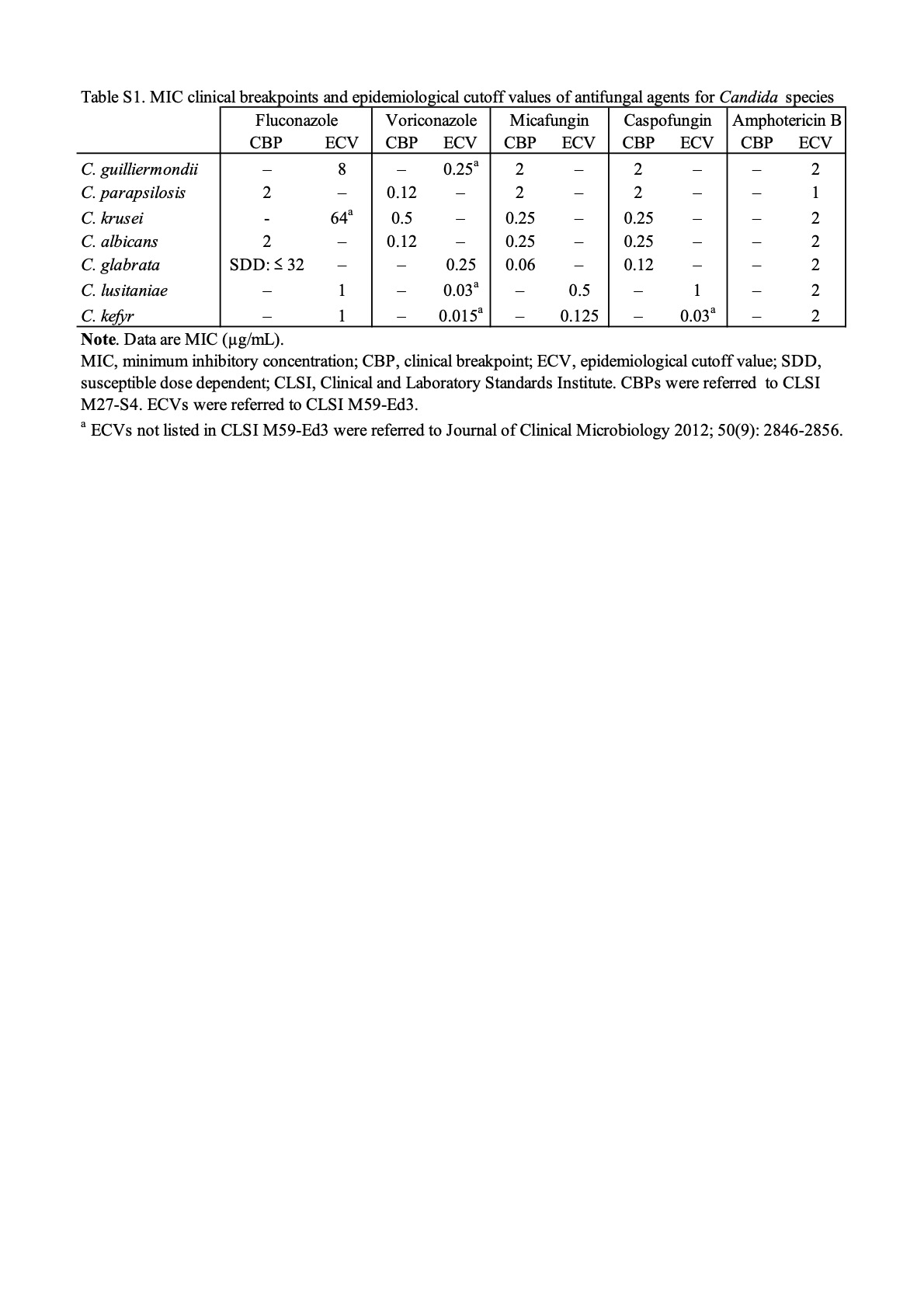

Supplement: myad056_Supplemental_Files [file myad056_supplemental_files.zip › mm-2023-0077-File005.jpg]
